# White matter changes in recovered COVID-19 patients: insights from DTI, DKI, and NODDI metrics

**DOI:** 10.3389/fneur.2025.1580262

**Published:** 2025-07-18

**Authors:** Manxi Yuan, Ruiqi Lu, Yu Liu, Hui Zhu, Hao Wang, Jingzhi Wang, Yang Song, Lei Yang, Mingzhong Xiao

**Affiliations:** ^1^The First School of Clinical Medicine, Hubei University of Chinese Medicine, Wuhan, China; ^2^School of Laboratory Medicine, Hubei University of Chinese Medicine, Wuhan, China; ^3^Hubei Provincial Hospital of Traditional Chinese Medicine, Wuhan, China; ^4^Afffliated Hospital of Hubei University of Chinese Medicine, Wuhan, China; ^5^Institute of Liver Diseases, Hubei Key Laboratory of the Theory and Application Research of Liver and Kidney in Traditional Chinese Medicine, Hubei Provincial Hospital of Traditional Chinese Medicine, Wuhan, China; ^6^Hubei Province Academy of Traditional Chinese Medicine, Wuhan, China; ^7^Ministry of Education and State Key Laboratory of Environmental Health (Incubating), School of Public Health, Tongji Medical College, Huazhong University of Science and Technology, Wuhan, China

**Keywords:** COVID-19, neurological sequelae, diffusion kurtosis imaging, diffusion tensor imaging, neurite orientation dispersion and density imaging, white matter

## Abstract

**Background:**

COVID-19 affects not only the respiratory system but also the central nervous system, resulting in symptoms such as anosmia and confusion. Understanding the long-term neurological effects of COVID-19 is critical for comprehensive patient care and management.

**Purpose:**

To study the long-term neurological effects of COVID-19, focusing on changes in white matter structural complexity using advanced neuroimaging techniques.

**Methods:**

Thirty-eight participants including 22 recovered COVID-19 patients and 16 healthy controls, underwent MRI scans with T1-weighted, T2-weighted, and diffusion-weighted imaging. Advanced diffusion sequences, including diffusion tensor imaging (DTI), diffusion kurtosis imaging (DKI), and neurite orientation dispersion and density imaging (NODDI), were used to assess microstructural integrity.

**Results:**

Significant differences in DKI metrics were observed, particularly in mean kurtosis (MK) and radial kurtosis (RK). Reduced MK and RK values were observed in certain regions, particularly the right inferior fronto-occipital fasciculus (IFOF), indicating reduced structural complexity of the white matter. No significant differences in DTI and NODDI metrics or clinical and demographic characteristics were found between the groups.

**Conclusion:**

This study highlights the potential long-term neurological sequelae in recovered COVID-19 patients as evidenced by changes in white matter structural complexity. These findings underscore the importance of continued monitoring and tailored interventions to address neurological sequelae as part of the post-COVID-19 recovery process.

## Introduction

It is well known that severe acute respiratory syndrome coronavirus 2 (SARS-CoV-2) attacks the lungs, subsequently causing viral pneumonia. However, SARS-CoV-2 also affects the central nervous system (CNS) through direct and/or indirect impacts (1), manifesting in symptoms such as sleep disturbances, anosmia, confusion, headaches, dizziness, and muscle pain, particularly in severe patients (2–4). Research indicates that SARS-COV-2 may enter the CNS through the hematogenous or retrograde neuronal route (5). Evidence of SARS-CoV-2 in the cerebrospinal fluid (CSF) confirms its capability to breach the blood–brain barrier, infiltrating neural tissues and causing cell damage (6). The virus likely accesses the brain via the olfactory nerve, spreading to interconnected regions, which may explain the common loss of smell observed in patients (7–9). In addition, the COVID-19 pandemic was linked with the increased global prevalence of major depression and anxiety (10), likely triggered by fear and anxiety related to COVID-19 as well as social isolation, resource scarcity, economic downturns, and increased unemployment due to socioeconomic changes (11). Research suggests that social isolation can disrupt the hypothalamic–pituitary–adrenal axis, leading to excessive arousal and insomnia, thereby exacerbating sleep disorders (12). Notably, coronaviruses also possess neurotropic properties (13), inducing inflammatory responses in neural tissues and alterations in neurotransmitters that contribute to mood and anxiety disorders (14).

Advanced neuroimaging techniques, including diffusion tensor imaging (DTI), diffusion kurtosis imaging (DKI), and neurite orientation dispersion and density imaging (NODDI), are sensitive tools for assessing the microstructural integrity of neuronal fiber bundles. DTI is utilized for its ability to reveal the Gaussian diffusion of water molecules, which helps in assessing the basic structural integrity of white matter. Despite its widespread use, the limitations of DTI in capturing complex fiber configurations are well-documented (15). DKI is a non-invasive diffusion imaging technique based on a fourth-order three-dimensional fully symmetric non-Gaussian distribution model, offering a more nuanced view of brain tissue microstructures to overcome the aforementioned limitations (16, 17). NODDI provides detailed insights into neurite architecture, surpassing conventional diffusion metrics by estimating neurite volume fraction and orientation dispersion (18, 19). This sophisticated approach enables a more comprehensive analysis of the non-Gaussian properties of water molecule diffusion within white matter (20–22).

In this study, we used the above advanced neuroimaging techniques to explore the neurological impacts of COVID-19, aiming to provide a comprehensive assessment of how the virus affects brain structure and function and correlate these findings with the diverse neurological symptoms.

## Materials and methods

### Study participants

The COVID-19 recovered patients were recruited from the Hubei Provincial Hospital of Traditional Chinese Medicine, and healthy control participants were recruited from the hospital’s health check-up center. The study group included 31 individuals who recovered from COVID-19 and 17 healthy controls, totaling 48 participants. All participants underwent follow-up at the Hubei Provincial Hospital of Traditional Chinese Medicine from November 2020 to March 2021.

The study was approved by the Ethics Committee of Hubei Provincial Hospital of Traditional Chinese Medicine (Approval Number: HBZY2020-C01-01). All participants, or their legally authorized representatives, provided written informed consent prior to participation.

Participants in the recovered from COVID-19 group were required to meet all the following criteria: (1) confirmed COVID-19 infection between November 2019 and February 2020, verified by reverse transcription polymerase chain reaction (RT-PCR) or chest computed tomography (CT) findings indicative of COVID-19 pneumonia; (2) age ≥18 years; and (3) underwent magnetic resonance imaging (MRI) between November 2020 and March 2021 approximately 1 year following recovery, meeting clinical indications and demonstrating the ability to comply with imaging procedures. The exclusion criteria were as follows: (1) history of progressive CNS disorders, including Alzheimer’s disease, Parkinson’s disease, amyotrophic lateral sclerosis, frontotemporal dementia, and dementia with Lewy bodies; (2) incomplete brain MRI datasets; or (3) contraindications to MRI.

In the experimental group of this study, 13/31 individuals (41.9%) exhibited neurological symptoms, including headache (18.18%), visual disturbances (4.55%), fatigue (59.09%), and insomnia (40.91%).

**Figure 1:**
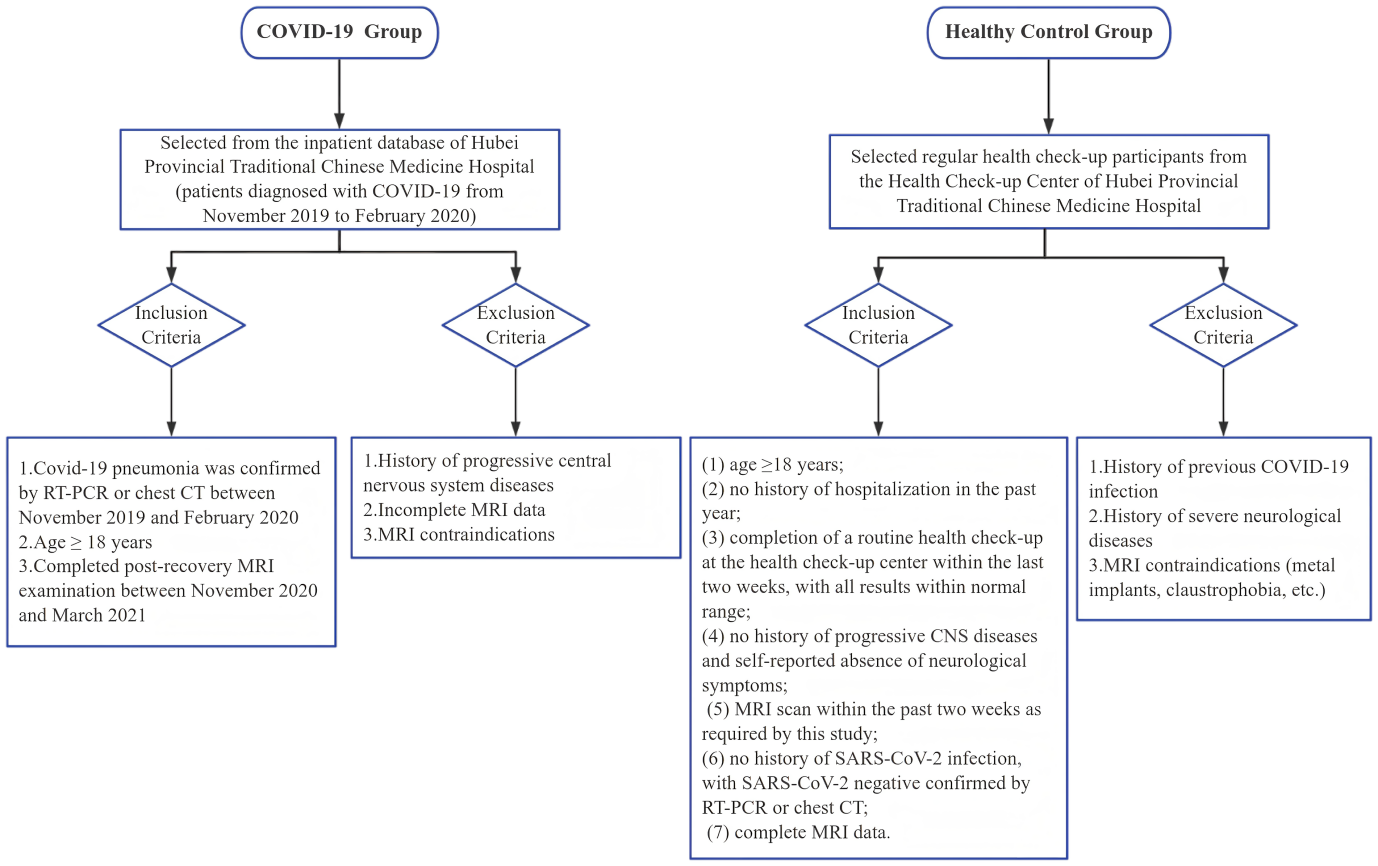
Flowchart of participant recruitment.

The control group was recruited from individuals who underwent routine health check-ups at the Health Check-up Center of Hubei Provincial Hospital of Traditional Chinese Medicine. The inclusion criteria for the control group included: (1) age ≥18 years; (2) no history of hospitalization in the past year; (3) completion of a routine health check-up at the health check-up center within the last 2 weeks, with all results within normal range; (4) no history of progressive CNS diseases and self-reported absence of neurological symptoms; (5) MRI scan within the past 2 weeks as required by this study; (6) no history of SARS-CoV-2 infection, with SARS-CoV-2 negative status confirmed by RT-PCR or chest CT; and (7) complete MRI data. The exclusion criteria included the following: (1) history of previous COVID-19 infection; (2) history of progressive CNS disease; and (3) MRI contraindication. All control participants underwent MRI scanning and routine blood tests and completed the required questionnaires (Figure 1).

Clinical characteristics and laboratory data of all participants were collected. Demographic and clinical information such as age, sex, medical history, height, and weight were systematically recorded. Comprehensive blood tests were performed, including measurements of white blood cell count (WBC), lymphocyte count (LYM#), granulocyte count (GRAN#), lymphocyte and granulocyte percentages (LYM%, GRAN%), neutrophil/lymphocyte ratio (NLR), red blood cell count (RBC), hemoglobin (HGB), and platelet count. Psychometric evaluations along with olfactory and gustatory rating scales were administered at the time of MRI scanning to assess the cognitive and sensory functions of the participants.

### MRI acquisition

All MRI data were acquired at the Department of Radiology, Hubei Provincial Hospital of Traditional Chinese Medicine, using a 3.0 Tesla MRI scanner (Siemens MAGNETOM Skyra XA60, Germany) equipped with a 32-channel head coil. The scanning protocol included T1-weighted imaging (T1WI), T2-weighted imaging (T2WI), fluid-attenuated inversion recovery (FLAIR), and diffusion-weighted imaging (DWI). The DWI acquisition parameters were as follows: repetition time (TR)/echo time (TE) = 5,400/92 ms; field of view = 224 × 224 mm; matrix = 112 × 112; 40 axial slices; voxel size = 2 × 2 × 3 mm; bandwidth = 1,654 Hz/pixel; b-values = 1,000 and 2,000 s/mm^2^; 64 diffusion gradient directions per b-value; and 10 b0 images. All participants were scanned in the supine position with soft padding placed between the head and the coil to minimize motion artifacts.

All MRI data underwent rigorous quality control procedures. Prior to preprocessing, brain MRI data of each participant were independently reviewed by two experienced neuroradiologists. The review involved interpreting T1WI, T2WI, FLAIR, and DWI sequences and evaluating whether image quality and scanning parameters met the study criteria. In cases of disagreement or uncertainty regarding image quality or eligibility, a third senior neuroradiologist provided the final decision. Based on these evaluations, 10 participants were excluded from the analysis for the following reasons: (1) three participants were diagnosed with meningiomas; (2) four participants had incomplete data, having undergone only T1WI, T2WI, and FLAIR sequences; (3) two participants sustained cranial trauma during rehabilitation, including one from the control group; and (4) the MRI volume of one participant did not meet the study requirements.

### Image analysis

The *dcm2niix* tool was used to convert raw imaging data into NIfTI format, and image quality was visually inspected using *MRIcroGL*. DWI data preprocessing, including denoising and artifact correction, was performed using *MRtrix*. The dwifslpreproc command in *MRtrix* was used to correct motion and eddy current distortions, with subsequent adjustment of b-vectors based on the estimated transformations. To address intensity inhomogeneities introduced by scanner bias fields, N4 bias field correction was applied using *ANTs*. Brain extraction was performed on the initial b0 image using the Brain Extraction Tool (*BET*) from the *FSL* suite to generate a brain mask for subsequent tensor estimation. Diffusion tensor estimation was conducted using the dwi2tensor command in *MRtrix*, from which standard diffusion metrics were derived, including fractional anisotropy (FA), mean diffusivity (MD), radial diffusivity (RD), and axial diffusivity (AD). For advanced diffusion modeling, DKI was implemented using the *DIPY* toolbox. The dkifit function was used to compute kurtosis metrics, including mean kurtosis (MK), axial kurtosis (AK), and radial kurtosis (RK). To further explore microstructural complexity, neurite orientation dispersion and density imaging (NODDI) modeling was performed using the *AMICO* framework, which enabled efficient estimation of microstructural indices such as neurite density index (NDI), orientation dispersion index (ODI), and the cerebrospinal fluid (CSF) volume fraction. Notably, the CSF fraction was excluded from white matter analyses due to its distinct biophysical characteristics.

### TBSS analysis

Tensor metrics were analyzed using the tract-based spatial statistics (TBSS) approach within the FSL framework with the following steps: (1) Alignment and averaging: Individual FA maps were aligned to a standard FA template through both linear and nonlinear registration methods. This was followed by the generation of a mean FA map that represents the averaged diffusion properties of all study participants. (2) Skeletonization: The mean FA map was skeletonized by applying a threshold of 0.2, which eliminated peripheral voxels that do not represent principal white matter tracts, thus ensuring focus on regions with FA values greater than 0.2 for further analysis. (3) Projection: The individual FA maps that had undergone registration were then projected onto the skeletonized mean FA map. This projection facilitated the creation of individual skeletonized FA maps, which are crucial for comparing microstructural integrity across the cohort. (4) Extension to other metrics: Utilizing the TBSS NonFA tool, additional diffusion metrics including MD, AD, RD, MK, AK, RK, NDI, and ODI were also projected onto the established FA skeleton.

### Statistical analysis

The microstructural metrics derived from the TBSS procedure were subjected to statistical analysis using the Randomize tool in FSL. A two-sample *t*-test, controlling for sex and age, was conducted with 5,000 permutations to enhance statistical validity. To address the issue of multiple comparisons, threshold-free cluster enhancement was used, setting a significance threshold of *p* ≤ 0.05. Statistically significant differences were identified and are detailed in Tables 1, 2.

**Table 1:** *T*-test for voxel differences in different regions (MK).

Cluster-ID	Brain region(s)	MNI peak coordinates (mm)			*P*	Cluster size
		X	Y	Z		
10	Inferior fronto-occipital fasciculus R	28	−8	25	0.02	14,793
9	Corticospinal tract L	−10	−72	−34	0.041	1964
8	Corticospinal tract R	17	−70	−38	0.044	1786
7	Inferior fronto-occipital fasciculus L	−19	−86	9	0.042	1,620
6	Inferior longitudinal fasciculus L	−38	−62	−12	0.049	126
5	Superior longitudinal fasciculus L	−35	−68	27	0.049	85
4	Anterior thalamic radiation L	−12	−20	14	0.049	64
3	Superior longitudinal fasciculus L	−38	−42	25	0.049	31
2	Inferior longitudinal fasciculus L	−32	−70	16	0.049	26
1	Anterior thalamic radiation L	−19	−36	7	0.049	24

**Table 2:** *T*-test for voxel differences in different regions (RK).

Cluster-ID	Brain region(s)	MNI peak coordinates (mm)			*P*	Cluster size
		X	Y	Z		
4	Inferior fronto-occipital fasciculus R	28	−15	18	0.017	12,594
3	Inferior longitudinal fasciculus L	−40	−53	37	0.038	4,152
2	Anterior thalamic radiation L	−18	10	8	0.044	622
1	Corticospinal tract L	−28	−32	49	0.046	427

## Results

### Demographics and clinical characteristics

The final study group included 38 participants, comprising 22 individuals recovered from COVID-19 and 16 healthy controls. The demographic and clinical characteristics of the two groups are summarized in Table 3. No significant differences were observed in sex distribution between groups, with males accounting for 54.55% in the COVID-19 group and 37.5% in the control group (*p* = 0.342). Similarly, there were no statistically significant differences in age or body mass index between the two groups. The prevalence of hypertension (22.73% vs. 25.00%, *p* = 1.000) and diabetes mellitus (9.09% vs. 12.50%, *p* = 1.000) was also comparable between the patient and control groups.

**Table 3:** Demographics of recovered COVID-19 patients and healthy controls.

Demographics information	Patients	Healthy controls	t/Z/χ2	*P*
*n*	22	16		
Sex (*n*, %)				0.342
Male	12 (54.55)	6 (37.5)		
Female	10 (45.45)	10 (62.5)		
Age, years (mean±SD)	53.64±11.47	52±6.87	6.763	0.615
BMI (mean±SD)	24.85±2.52	23.69±2.65	0.016	0.179
Hypertension (*n*, %)				1.000
Yes	5 (22.73)	4 (25)		
No	16 (72.73)	12 (75)		
Diabetes (*n*, %)				1.000
Yes	2 (9.09)	2 (12.5)		
No	19 (86.36)	14 (87.5)		

Table 4 presents the biochemical results of the participants. There were no significant differences in WBC, LYM#, GRAN#, NLR, LYM%, GRAN%, RBC, HGB, platelet count, or AST between the two groups (all *p* > 0.05).

**Table 4:** Biochemical and hematological results of recovered COVID-19 patients and healthy controls [(x ± s)/M (IQR)].

Biochemical and hematological results	Patients (*n* = 22)	Healthy controls (*n* = 16)	t/Z/χ2	*P*
WBC	5.59±1.37	5.84±1.27	−1.454	0.156
LYM#	1.93±0.52	1.75±0.29	1.165	0.252
GRAN#	3.12±1.14	4.00±1.66	−1.865	0.071
NLR	1.71±0.73	2.36±1.18	−2.032	0.05
LYM%	35.36±8.70	29.22±7.94	2.083	0.045
GRAN%	54.74±8.92	60.71±7.62	−2.016	0.052
RBC	4.73±0.52	4.56±0.40	0.384	0.704
HGB	143±15.55	143.36±14.23	−0.493	0.626
platelet	218.55±50.49	216.95 (23.90)	−0.068	0.946
AST	25.77 (10.50)	24.91±7.67	−0.181	0.857

The mental health assessment scale scores and the prevalence of olfactory and taste changes are shown in Table 5. No significant differences were found in the scores of the General Health Questionnaire-12 (GHQ-12), Post-traumatic Stress Disorder Checklist-5 (PCL-5), Generalized Anxiety Disorder-7 (GAD-7), or Patient Health Questionnaire-9 (PHQ-9) between the patient and control groups. A higher proportion of patients reported olfactory changes compared with the controls (40.00% vs. 12.50%), although the difference was not statistically significant (*p* = 0.214). The prevalence of taste changes was similar between the two groups (20.00% vs. 28.57%, *p* = 0.633).

**Table 5:** Mental health assessment scale and olfactory/taste changes.

Mental health assessment scale and olfactory/taste changes	Patients	Healthy controls	t/Z/χ2	*P*
GHQ-12	3.58 (2.75)	1.86±1.46	−0.985	0.325
PCL-5	11.42±7.03	14.00±12.17	−1.627	0.116
GAD-7	2.75 (4.00)	4.57±5.00	−1.881	0.06
PHQ-9	4.58 (6.25)	3.14±2.04	−0.860	0.390
Olfactory changes	8 (40.00%)	1 (12.50%)		0.214
Taste changes	4 (20.00%)	2 (28.57%)		0.633

### Differences in DKI between the two groups

Significant differences were detected in DKI metrics (MK and RK) between the COVID-19 and control groups, with significant reductions observed in specific brain regions for the COVID-19 group (*p* < 0.05). However, no significant differences were found between the two groups regarding DTI metrics (FA, MD, RD, and AD) and NODDI metrics (NDI and ODI). Moreover, the T2/FLAIR signals demonstrated multiple regions of elevated signal intensity within the subcortical, thalamus, and basal ganglia regions in both groups. However, no notable differences were observed between the two groups.

**Figure 2:**
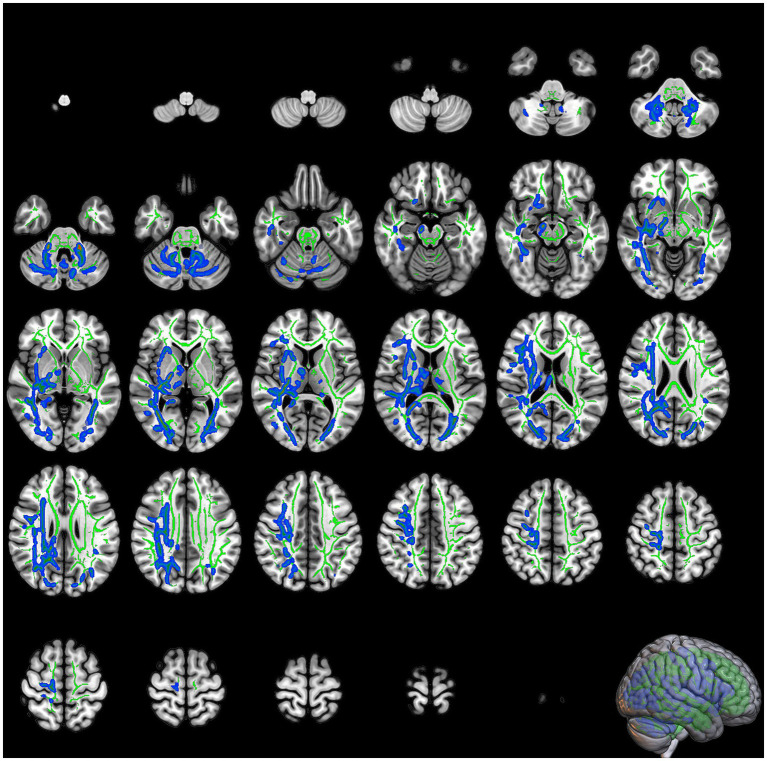
The slices of significant clusters in mean kurtosis (MK). The background is the MNI152 standard brain map, FA indicates skeleton, green represents white matter skeleton, and blue indicates regions of significant difference.

DKI analysis revealed that patients diagnosed with COVID-19 exhibited significant reductions in MK within the right IFOF, bilateral corticospinal tract, left IFOF, left inferior and superior longitudinal fasciculus (SLF), and left anterior thalamic radiation (Table 1; Figure 2). The most significant reduction in MK was observed in the right IFOF (cluster size: 14793 voxels, *p* = 0.02), followed by the left corticospinal tract (cluster size: 1964 voxels, *p* = 0.041).

**Figure 3:**
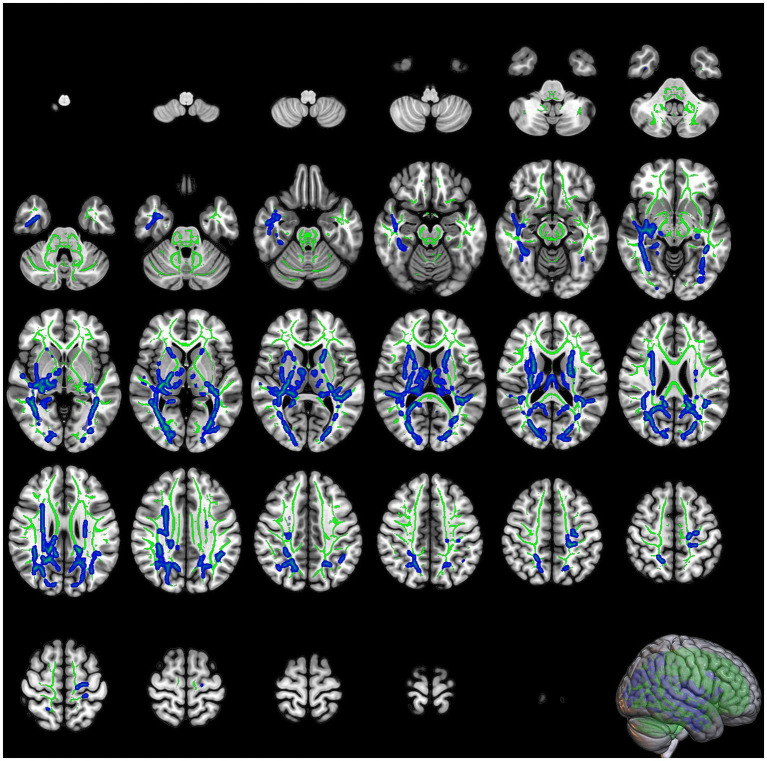
The slices of significant clusters in radial kurtosis (RK). The background is the MNI152 standard brain map, FA indicates skeleton, green represents white matter skeleton, and blue indicates regions of significant difference.

Significant reductions in RK were observed in the right IFOF, left inferior longitudinal fasciculus (ILF), left anterior thalamic radiation, and left corticospinal tract in the COVID-19 group compared with the control group (Table 2; Figure 3). The most prominent reduction in RK was found in the right IFOF (cluster size: 12594 voxels, *p* = 0.017), followed by the left ILF (cluster size: 4152 voxels, *p* = 0.038).

## Discussion

In addition to the typical respiratory symptoms, approximately 30% of COVID-19 survivors experience neurological sequelae, including cognitive impairment, loss of smell, and motor dysfunction (23). Autopsy studies have shown that COVID-19 patients exhibit congestion and edema in brain tissue, and SARS-CoV nucleic acids have been detected in cerebrospinal fluid (24, 25). Among the areas most commonly affected by SARS-CoV-2 damage are the insula and thalamic radiations (26). The underlying mechanism suggests that SARS-CoV-2 can invade the central nervous system through neural cells expressing the ACE2 receptor (27). Notably, some patients still exhibit persistent white matter microstructural changes even after acute symptoms have subsided, suggesting that the virus may lead to long-term neurological damage through latent neuroinflammation or axonal injury. Research by Sherif et al. shows that MD remains elevated during the recovery phase after other COVID-19 symptoms have subsided (28). Three to four months after SARS-CoV-2 infection, the volumes of gray matter and white matter are significantly increased (29).

Research indicates that the combination of DKI, NODDI, and DTI can provide more comprehensive assessment of brain microstructure. For instance, in patients with traumatic brain injury, DKI and NODDI metrics demonstrate greater sensitivity during the acute phase. Moreover, only NODDI can detect subtle changes in parallel fiber structures such as those in the corpus callosum (21). Therefore, this study simultaneously employed DKI, NODDI, and DTI to comprehensively assess the long-term effects of COVID-19 on brain microstructure. The study results indicate that approximately 1 year after infection, subjects in the COVID-19 recovery group still showed a significant reduction in MK and RK in certain brain regions. MK is proportional to the heterogeneity and complexity of brain microstructure (30), and a reduction in MK indicates the loss of cellular structures (31), such as the total volume of white matter, the total volume of myelinated fibers, total length, length density, volume density, and average diameter. Radial diffusion of water molecules is more sensitive (32), and RK is highly sensitive to the integrity of axons and myelin. A reduction in RK indicates a weakening of the restriction in water molecule diffusion (33). The results suggest that SARS-CoV-2 selectively induces white matter microstructural degeneration, with neuronal and myelin damage in the corresponding brain regions, which is consistent with autopsy findings (34). In animal experiments, susceptible mice exhibited demyelinating lesions in the central nervous system (35). It is worth noting that in this study, no significant changes were observed in the DTI and NODDI indices. DTI simplifies the process of water molecule diffusion, such as MD and RD, which can only describe cases based on Gaussian diffusion. The kurtosis parameter in DKI, however, reflects the complexity of finer microstructural details (36). In late fetal brain development, MK decreases with the increase in cortical complexity, but FA shows no significant change (37). In patients with amyotrophic lateral sclerosis, MK and RK in the fornix are decreased, while DTI indices show no significant changes (38). In glioma grading, MK has higher diagnostic value than MD (39). In detecting neural system development and pathological changes, DKI shows higher sensitivity and specificity. NODDI can theoretically analyze the microstructure of white matter specifically (40). However, SARS-CoV-2 triggers mitochondrial damage, and complex changes occur in cell physiology (41). thereby disrupting the basic assumptions of the NODDI model, resulting in deviations in NDI and ODI (20). In contrast, MK and RK can directly reflect axon-myelin complex damage. Consistent with the conclusions of the current study, the study by Churchill et al. reported that MK increased in patients 4–5 months after COVID-19 infection, but FA, RD, NDI, and ODI showed no significant differences (42). Similarly, Van et al. showed that during hospitalization, there were no significant changes in NDI in COVID-19 patients, ODI decreased 3 months later, and after 12 months, no changes were observed in the above indicators (43). The differences in the results may be related to the basic assumptions of the NODDI model: its specificity depends on the premises of the specific model. However, in clinical settings, different pathological mechanisms often coexist, making it difficult for a single model to fully capture heterogeneous pathological changes (44). The differences in results may also stem from variations in the timing of data collection, different stages of post-infection evaluation, and differences in recovery and pathological heterogeneity, such as individual variations in the recovery process and injury severity. It may also reflect specific age-related associations, as changes in indicators are closely related to the age of the subjects (18). The age distribution of the group included in this study may exacerbate the variability of the results.

Our results also show that about a year after recovery, there are still white matter microstructural changes in the corresponding brain regions, indicating that SARS-CoV-2 has long-term effects on the nervous system. The immune response triggered by acute infection leads to demyelination in the brain, resulting in delayed brain injury (45, 46). Approximately 1 year after infection, COVID-19 patients show a significant decrease in intracellular water fraction in white matter regions such as the corona radiata, corpus callosum, and SLF, suggesting a reduction in axonal density or impairment of neural integrity (47). Our results are largely consistent. In the COVID-19 recovery group, MK was significantly reduced in the right IFOF, bilateral corticospinal tract, left IFOF, left inferior and SLF, and left anterior thalamic radiation. RK was significantly reduced in the right IFOF, left ILF, left anterior thalamic radiation, and left corticospinal tract. COVID-19 patients and individuals in the recovery group commonly experience fatigue and weakness (48). The intensity of fatigue is significantly correlated with the axonal integrity of the corticospinal tract and thalamic radiation. When the myelination of the motor cortex bundle is insufficient or damaged, there is a certain degree of deficiency in the patients’ executive function and attention (48). Long-range fibers (such as the fronto-occipital fasciculus, thalamic radiation, SLF, and ILF) have relatively longer lengths and a higher membrane-to-cytoplasm ratio. These fibers also have lower blood flow but higher metabolic demands, making them more susceptible to damage (49, 50).

Furthermore, from a structural MRI perspective, neuroimaging studies in severe COVID-19 patients often reveal medial temporal lobe involvement, multifocal white matter hyperintensities on FLAIR with non-confluent lesions, varying contrast enhancement, and hemorrhagic features, as well as diffuse or isolated microbleeds (2), likely due to demyelination, endothelial injury, and cytokine storms (51). However, most participants in our cohort had mild to moderate symptoms and were not critically ill, and no significant abnormalities were detected on conventional MRI (e.g., FLAIR/T2), suggesting that neuropathological processes may not be prominent in less severe cases. While white matter changes appear to be a common consequence of COVID-19, variations in affected regions may be influenced by differences in clinical severity, demographics, or time since infection. In our study, there was no significant difference in baseline age between the COVID-19 (53.64 ± 11.47 years) and control (52 ± 6.87 years) groups. Both groups exhibited nonspecific T2/FLAIR hyperintensities in the subcortical, thalamic, and basal ganglia regions, which may reflect age-related microvascular, inflammatory, or metabolic changes rather than COVID-19-specific effects. The small sample size (22 COVID-19, 16 controls) may also have limited statistical power, underscoring the need for larger studies to confirm these observations.

At the same time, there were no statistically significant differences in the GHQ-12, PCL-5, GAD-7, or PHQ-9 scores between the patient and control groups. Compared with the control group, the proportion of individuals with olfactory changes was relatively higher in the patient group, while the rate of taste changes was similar between the two groups. These differences were not statistically significant. Olfactory dysfunction is one of the most frequently reported symptoms during the acute phase of COVID-19, with a prevalence ranging from 47 to 62% (52). On a mechanistic level, both SARS-CoV and SARS-CoV-2 are known to exhibit neurotropism via the ACE2 receptor, which is expressed in neurons (9, 53, 54). Studies in K18-hACE2 mice showed that SARS-CoV spreads transneuronally from the olfactory bulb to the brain, leading to neuronal loss and transneuronal dissemination (55). The mechanism may underlie both the early olfactory symptoms and the broader structural brain changes observed in post-COVID patients. Prior imaging studies have implicated several tracts involved in olfactory processing—including the corticospinal tract, arcuate fasciculus, IFOF, thalamus-parietal fasciculus, thalamus-occipital fasciculus, and posterior corpus callosum—as being affected (56). However, in our cohort, olfactory function assessments revealed no significant differences between groups, and MRI showed no observable abnormalities in the olfactory sulcus or bulb. This may be due to spontaneous recovery of olfactory function over time, especially considering that our participants were approximately 1 year post-infection. Previous studies reported olfactory recovery rates of 85–95% within 6 months (57, 58). The results showed no significant differences in neurological symptoms between the two groups, indicating that microscopic structural changes and clinical manifestations are not synchronized. The lack of symptomatic differences may also reflect limitations in sample size and the sensitivity of clinical assessment tools.

This study has several limitations: First, the possibility of pre-existing health biases cannot be ruled out. Due to the unexpected restrictions imposed by the pandemic, no baseline MRI data were collected from any of the patients prior to infection. The observed brain differences have a substantial probability of reflecting pre-existing neurobiological characteristics in the patient group (such as neurodegenerative risks or structural abnormalities), rather than COVID-19-specific changes. This fundamental limitation makes it impossible to distinguish the independent contributions of infection’s direct effects and baseline differences. Moreover, the small sample size, lack of acute phase imaging follow-up (such as MRI scans within 1 month post-infection), and absence of objective olfactory tests limit the tracking of longitudinal dynamic changes. While the control group was carefully selected, it may still introduce confounding bias due to the inclusion of asymptomatic individuals with past infections. Additionally, the lack of analysis of metabolic factors (such as cholesterol, HbA1c) and inflammatory markers (such as CRP) may have missed key covariates (59–61). Among the imaging results, only MK and RK showed significant differences, with other parameters not reaching statistical significance; therefore, the conclusions should be interpreted with caution. Notably, the separation between imaging changes and clinical symptoms requires careful interpretation. Three equally plausible explanations exist: (1) the viral-induced microstructural changes may precede the onset of symptoms; (2) pre-existing neurodevelopmental or neurodegenerative differences in the patient cohort may have predisposed individuals to both COVID-19 infection and subsequent neurological symptoms; or (3) these findings represent incidental differences unrelated to infection. Given the absence of baseline data, this study cannot determine the relative contributions of these mechanisms. While bias was reduced through strict inclusion criteria and careful control group selection, the strong likelihood that our results represent pre-existing neural differences must be recognized as a fundamental limitation.

Overall, this study suggests that only MK and RK have unique value in detecting white matter microstructural damage in COVID-19 recovered patients. Their sensitivity arises from the precise quantification of microstructural heterogeneity (such as axonal density, myelin integrity) and changes in the cellular membrane microenvironment. Conventional DTI or NODDI measures are unable to detect these changes, and although efforts were made to reduce bias through strict inclusion criteria and careful selection of control groups, the conclusions should be interpreted with caution. Future research using longitudinal and multimodal approaches will be essential to fully elucidate the long-term effects of SARS-CoV-2 on brain structure and function.

## Data Availability

The original contributions presented in the study are included in the article/supplementary material, further inquiries can be directed to the corresponding authors.

## Glossary

MRI: Magnetic Resonance Imaging
DTI: Diffusion Tensor Imaging
DKI: Diffusion Kurtosis Imaging
NODDI: Neurite Orientation Dispersion and Density Imaging
MK: Mean Kurtosis
RK: Radial Kurtosis
AK: Axial Kurtosis
FA: Fractional Anisotropy
MD: Mean Diffusivity
RD: Radial Diffusivity
AD: Axial Diffusivity
NDI: Neurite Density Index
ODI: Orientation Dispersion Index
CNS: Central Nervous System
CSF: Cerebrospinal Fluid
SARS-CoV-2: Severe Acute Respiratory Syndrome Coronavirus 2
RT-PCR: Reverse Transcription-polymerase Chain Reaction
CT: Computed Tomography
WBC: White Blood Cell
LYM#: Lymphocyte Count
GRAN#: Granulocyte Count
LYM%, GRAN%: Lymphocyte and Granulocyte Percentages
NLR: Neutrophil/Lymphocyte Ratio
RBC: Red Blood Cell Count
HGB: Hemoglobin
DWI: Diffusion-weighted Imaging
BMI: Body Mass Index
TBSS: Tract-based Spatial Statistics
GHQ-12: General Health Questionnaire-12
PCL-5: Post-traumatic Stress Disorder Checklist-5
GAD-7: Generalized Anxiety Disorder-7
PHQ-9: Patient Health Questionnaire-9
IFOF: Inferior Fronto-occipital Fasciculus
SLF: Superior Longitudinal Fasciculus
ILF: Inferior Longitudinal Fasciculus
ACE2: Angiotensin-converting Enzyme 2
